# Measuring food access using least-cost diets: Results for global monitoring and targeting of interventions to improve food security, nutrition and health

**DOI:** 10.1016/j.gfs.2024.100771

**Published:** 2024-06

**Authors:** Jessica K. Wallingford, Saskia de Pee, Anna W. Herforth, Sabrina Kuri, Yan Bai, William A. Masters

**Affiliations:** a Friedman School of Nutrition Science and Policy, Tufts University, Boston MA, USA; b United Nations World Food Programme, Rome, Italy; c Development Data Group, The World Bank, Washington DC, USA; d Food Prices for Nutrition Project, Tufts University, Boston MA, USA; e Department of Economics, Tufts University, Medford MA, USA

**Keywords:** Least-cost diets, Affordability, Food security, Global monitoring, Food system intervention

## Abstract

Benchmark diets using the most affordable locally available items to meet health and nutrition needs have long been used to guide food choice and nutrition assistance. This paper describes the result of recent innovations scaling up the use of such least-cost diets by UN agencies, the World Bank, and national governments for a different purpose, which is monitoring food environments and targeting systemic interventions to improve a population's access to sufficient food for an active and healthy life. Measuring food access using least-cost diets allows a clearer understanding of where poor diets are caused by unavailability or high prices for even the lowest-cost healthy foods, insufficient income or other resources to acquire those foods, or the use of other foods instead due to reasons such as time use and meal preparation costs, or cultural factors such as taste and aspirations. This paper reviews the data, methods and results that have led to official FAO and the World Bank adoption of cost and affordability metrics for global monitoring, and the parallel use of similar methods to guide interventions in country studies led by the World Food Programme with partner agencies across Africa, Asia and Latin America. We conclude by summarizing how increasing availability of food price data, matched to food composition and dietary requirements, allows analysts to use recently developed software tools for least-cost diet assessment to improve food access in a wide range of settings.

## Highlights

• Least-cost healthy diets are used to monitor food access and guide interventions.
• Indicators of cost per day and affordability help reveal causes of poor nutrition.
• Use for global monitoring provides international benchmarks.
• Use within countries helps target actions to improve affordability.
• Results show impacts of new diet cost indicators and the path to further improvements.

## 1 Background and motivation: Why use least-cost diets to assess and guide interventions in food security and nutrition?

Least-cost diet indicators have recently been taken up by UN agencies, the World Bank, and national governments for assessment of food security and nutrition and targeting of food system interventions. Food security is a global priority, and its assessment has a long and distinguished history. Early approaches to assessing food security focused on total calories, beginning with a method introduced by Sukhatme (1961) using annual data from national food balance sheets together with a log-normal distribution of dietary energy intake and estimated minimum requirements to estimate the number of people in the world with average intake below what would be needed by a healthy population at reference levels of height, weight, and physical activity. The resulting Prevalence of Undernourishment (PoU) has been published by FAO since 1974, tracking the first global definition of food security as “*availability at all times of adequate world food supplies of basic foodstuffs*” (United Nations, 1975). In the 1990s, a more direct measurement method was introduced through surveys asking people if they skipped a meal, ate less than they wanted to, went to bed hungry, worried about food or had other similar experiences over the previous year, due to lack of resources to obtain their usual diet. This type of food security questionnaire has been used by the U.S. government since 1995 (USDA, 2024) and a version adapted for international comparisons known as the Food Insecurity Experience Scale (FIES) has been used for global monitoring since 2017 (FAO, 2023a).

The current and widely accepted definition of food security is ‘*when all people, at all times, have physical and economic access to sufficient, safe and nutritious food to meet their dietary needs and food preferences for an active and healthy life*’ (World Food Summit, 1996). Following the world food price spikes of 2008-09 and 2011-12, a variety of studies reviewed existing food security metrics and advocated for improved measurement to more fully reflect that definition (Herforth and Ahmed, 2015; Lele et al., 2016; Leroy et al., 2015); Herforth (2015) advocated for an indicator of “Cost of healthy diets” based on the prices of different food groups. This paper reviews the new approach developed in response to those concerns, guiding intervention using the least expensive locally available items for nutrient adequacy (de Pee et al., 2017) and monitoring food access using least-cost diets that would meet dietary guidelines globally (Herforth et al., 2020) and within countries by national government agencies in Ethiopia, Nigeria, Pakistan and elsewhere (Alemayehu et al., 2023; National Bureau of Statistics, 2024; Fatima et al., 2024).

The diet cost and affordability metrics described in this review can be traced back to George Stigler's Stigler (1945) introduction of the least-cost diet problem, defined as the set of locally available foods in quantities sufficient to meet a variety of dietary constraints at lowest total cost. The techniques used to compute least-cost diets for any given population at each place and time have evolved with changes in data availability and scientific knowledge on economic factors in malnutrition, for which the recent literature is surveyed in Masters et al. (2022) and summarized in a new textbook on *Food Economics: Agriculture, Nutrition and Health* (Masters and Finaret, 2024). Stigler's original diet problem considered lower bounds for just nine nutrients, while modern studies use both upper and lower bounds for over 20 essential nutrients with varying needs for specific populations (Deptford et al., 2017; Bai et al., 2022; Schneider et al., 2023), and also monitor the least expensive items by food group used for diet quality standards such as the Minimum Diet Diversity for Women (MDD-W) indicator (Masters et al., 2018), the EAT-*Lancet* reference diet (Hirvonen et al., 2020), national food-based dietary guidelines (Herforth et al., 2020; Alemayehu et al., 2023), and the global Healthy Diet Basket targets currently used for global and within-country monitoring (Herforth et al., 2022; FAO, 2023b; The World Bank, 2023a; National Bureau of Statistics, 2024).

Least-cost diets are useful for operationalizing the 1996 definition of food security, where ‘*economic access*’ is assessed by comparing the total cost per day of the least expensive locally available items to incomes available for food, in sufficient quantities ‘*to meet dietary needs*’ as represented by the nutrient or food group constraints included in the least-cost diet model. Least-cost diets are a modelled benchmark against which to compare actual food choice and diet quality, and identify where poor nutrition is caused by unavailability or unusually high prices for even the least-cost items in a healthy diet, by insufficient incomes to afford those options, or by displacement of that low-cost healthy diet by other foods for reasons such as time use and meal preparation costs, or tastes and aspirations. All three causes of poor nutrition could apply in each setting, with the least-cost diet benchmark revealing the degree to which a population faces unusually high prices, has insufficient income, or consumes an unhealthy diet for other reasons. Where prices for least-cost items are unusually high, locally-adapted innovations and investments in supply and distribution to improve availability and reduce diet costs would be needed. Where income available to buy food is insufficient, targeted social assistance and income growth to raise affordability would be needed. And where healthy diets are affordable but other factors limit use of healthy diets, changes in the food environment and food choice would be needed. The following section of this paper presents the data, methods and results of global monitoring, followed by a separate section on use of these methods to guide food system interventions within countries in Africa, Asia and Latin America.

## 2 Global monitoring: data sources, methods and results

Use of least cost diets for worldwide comparisons began in 2020, when the FAO introduced the cost and affordability of a healthy diet (CoAHD) suite of indicators developed by Herforth et al. (2020) to compute the number and percentage of people in each country unable to afford sufficient quantities of locally available foods to meet national dietary guidelines. Initial CoAHD results were introduced in the United Nations agencies’ annual flagship report on *The State of Food Security and Nutrition in the World* (FAO, IFAD, UNICEF, WFP and WHO, 2020; 2021). Demand for the indicator led to methodological refinements designed for global monitoring over time (Herforth et al., 2022), the results of which continued to be presented in annual flagship reports (FAO and UNICEF, 2022, FAO and UNICEF, 2023) and also published simultaneously by FAOSTAT (FAO, 2023b) and the World Bank (The World Bank, 2023b), and reproduced in other outlets such as Our World in Data (Ritchie et al., 2023) and the Food Systems Dashboard (Fanzo et al., 2020; Food Systems Dashboard, 2023).

Global monitoring of food access using the least expensive locally available items uses the Cost of a Healthy Diet (CoHD) indicator, defined as the lowest possible total cost per day needed to meet requirements for energy balanced among food groups as specified in dietary guidelines. Food quantities in each group for the purpose of global monitoring are specified in the Healthy Diet Basket (HDB) standard introduced in Herforth et al. (2022) for FAO, IFAD, UNICEF, WFP and WHO (2022). The HDB target reflects commonalities among national dietary guidelines as well as World Health Organization recommendations, specifying the need for 11 different items from six food groups. In this approach to global monitoring of access to healthy diets, the nutritional attributes of each item are captured by their food group, defined such that each country's least-cost diet meeting those food group requirements also meets almost all nutrient requirements (Herforth et al., 2022). Overall energy balance is specified at 2330 kcal, derived from the daily energy needs of the median healthy adult woman (not pregnant or breastfeeding) in the WHO reference population, which happens also to be almost exactly the average for all age-sex strata in that population. For diversity among and within food groups, the Healthy Diet Basket is specified as two starchy staples, three vegetables, two fruits, two animal-source foods, one item from the legumes, nuts and seeds category, and one item from the fats and oils category, with each quantity specified in energy terms so as to obtain the same quantity of nutrients and other bioactive compounds when substituting items with different water weight. Selecting the least expensive locally available items in each food group and summing up their total cost per day yields the CoHD (Herforth et al., 2023).

Computing the benchmark CoHD for countries across the world was made possible by a set of new data analysis techniques developed in a series of studies over the 2017–2022 period (Food Prices for Nutrition, 2023a), using retail prices at local markets matched to food composition data and nutritional requirements for health of a representative individual in each population of interest. Selection of the lowest-cost item is based on price per calorie of items with each nutritional attribute, starting with a pilot study in Ghana and Tanzania introducing a Cost of Diet Diversity (CoDD) indicator for least-cost items in at least five of ten specific food groups to reach the MDD-W threshold of dietary diversity, contrasted to the Cost of Nutrient Adequacy (CoNA) indicator for least-cost items to stay within upper and lower bounds for essential nutrients (Masters et al., 2018). A set of ten national FBDGs representing all world regions were used for the first global measurement of CoHD (Herforth et al., 2020), and the common elements among them and other FBDGs were then used to form a single Healthy Diet Basket target suitable for global monitoring (Herforth et al., 2022).

Food prices used in the calculation of diet costs for global monitoring by the FAO and the World Bank come from each country's national statistical organization through the International Comparison Program (ICP), reporting nationally-representative average prices for widely sold items in a reference year (The World Bank, 2023c). Initial calculations of CoHD in 2020–2023 were based on prices in 2017, with each of 172 countries reporting local availability and price for an average of 125 items from the ICP's global and regional list of over 700 internationally standardized items. Each country reports prices in their local currency, converted by ICP into international dollars at Purchasing Power Parity (PPP) exchange rates for household expenditure. International comparison of CoHD is made possible by these currency conversions, which are designed so that one PPP dollar can buy a similar level of all goods and services commonly used by households in each country. The purpose of ICP is to compute PPP exchange rates and thereby measure economic activity in real terms for each country and the world as a whole, after adjusting for inflation and differences in the value of local currencies. The ICP's central role in global economic statistics makes it the only platform through which almost all governments report nationally representative retail consumer prices with sufficiently standardized descriptions to identify the item's nutritional composition. This enables calculation of costs per day for almost all of the world's population, with two important limitations. A first concern is that the difficulty of international coordination means that prices are reported for a reference year with some delay, such as the prices for 2017 released in 2020, followed by prices for 2021 released in 2024 (The World Bank, 2023a). For global monitoring, that limitation is partially addressed by updating CoHD based on each country's average inflation for all food and non-alcoholic beverages as reported to the IMF and the FAO. A second concern is that, by definition, the ICP dataset is limited to items sold in more than one country, thereby omitting country-specific food items that might potentially enter a least-cost healthy diet. The purpose of the ICP requires international comparability, so reporting is limited to global and regional lists of the most widely consumed foods. These items are typically among the lowest-cost versions of each product, and countries that report prices for a larger number of items have generally included higher-cost versions or more premium products whose inclusion does not affect the lowest cost healthy diet, although countries that report prices for very few items have often omitted foods that actually are available and would be included in a least-cost diet leading to a systematic overestimate of CoHD (Bai et al., 2022, app. Figure S2 and Table S1; Headey et al., 2023). Both limitations are overcome by equipping national governments to use their own consumer price index item prices, which then permits within-country monitoring of monthly variation by region as done in Nigeria (National Bureau of Statistics, 2024) and elsewhere.

**Fig. 1:**
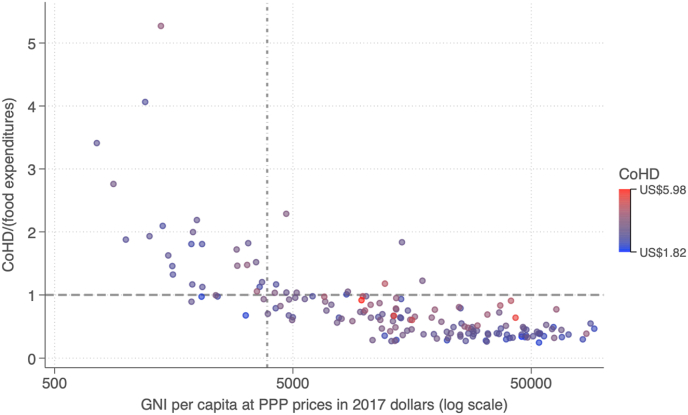
Ratio of the Cost of a Healthy Diet to average food expenditure per capita per day across countries, 2017 Note: Data shown are ratios of the cost of a healthy diet to national average food expenditures in 2017, downloaded from https://databank.worldbank.org/source/food-prices-for-nutrition. National income (GNI) data are from the World Development Indicators https://databank.worldbank.org/source/world-development-indicators. The dashed vertical line at US$3896 indicates the threshold between lower middle-income and upper middle-income economies according to World Bank country income classifications based on 2017 GNI per capita data, available at https://datahelpdesk.worldbank.org/knowledgebase/articles/906519-world-bank-country-and-lending-groups. CoHD, Cost of a Healthy Diet; PPP, purchasing power parity.

Estimating the number and percentage of people in each country who cannot afford a healthy diet is done by comparing CoHD to income available for food, using distributions of income or expenditure from national household surveys compiled by the Poverty and Inequality Platform of The World Bank (2023d). For global monitoring, the available data as of 2022 led to the threshold of unaffordability being defined as when CoHD cost more than 52 percent of a household's total income or expenditure. That threshold was based on the average food expenditure share of all households in low-income countries, from national accounts computed as part of the ICP database. This threshold had the advantage of being evidence-based and easily communicated but does not allow for variation in nonfood requirements. Updated global estimates to be released in 2024 will use newly released item prices from the ICP, and could also update the affordability method to use new country-specific data on nonfood expenditure. Using the current threshold of 52 percent of a household's income, an estimated 3.1 billion people in the world in 2017 could not afford a healthy diet (FAO, 2023b; The World Bank, 2023b). As shown in Fig. 1, CoHD is substantially above average food expenditures per capita per day in low- and lower-middle-income countries, because their actual consumption includes more low-cost starchy staples and other items than in the Healthy Diet Basket. In contrast, CoHD is below observed food spending in upper-middle and high-income countries, where people consume more expensive foods than the least-cost items needed for health.

**Fig. 2:**
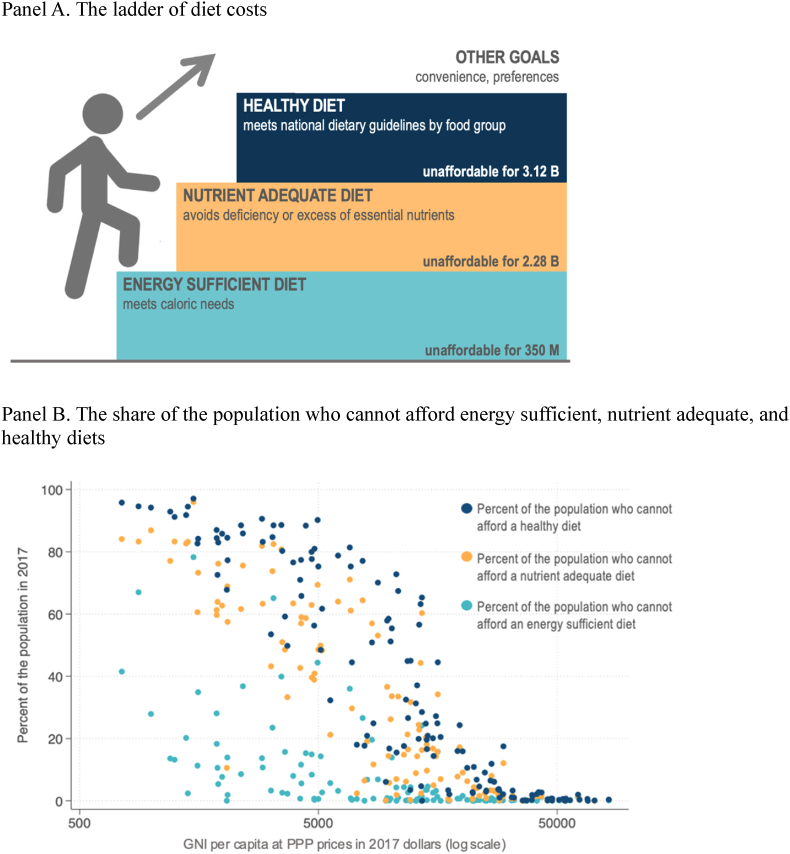
Affordability of energy sufficient, nutrient adequate, and healthy diets, 2017 Panel A. The ladder of diet costs Panel B. The share of the population who cannot afford energy sufficient, nutrient adequate, and healthy diets Note: The ladder of affordability infographic in Panel A was adapted from https://sites.tufts.edu/foodpricesfornutrition/. Affordability values in Panel A and data shown in Panel B are estimates for 2017, downloaded from https://databank.worldbank.org/source/food-prices-for-nutrition. National income (GNI) data are from the World Development Indicators https://databank.worldbank.org/source/world-development-indicators. PPP, purchasing power parity.

The affordability of meeting energy needs with different dietary patterns is shown in Fig. 2, starting with the schematic ladder of diet costs in Panel A. The first step for short-term survival would be to maintain energy balance with only the least expensive locally available starchy staple, which for the target of 2330 kcal/day at prices reported to ICP would have been unaffordable for 350 million people worldwide in 2017. A next step would be enough dietary diversity to stay within upper and lower bounds for essential nutrients, which would have been unaffordable for 2.3 billion people, and then the added cost of meeting food group targets specified in the Healthy Diet Basket makes CoHD unaffordable for 3.1 billion people, with variation among countries at each level of national income shown in Panel B of Fig. 2.

## 3 Targeting of interventions: data sources and methods from WFP's Fill the Nutrient Gap

Fill the Nutrient Gap (FNG) analyses use least-cost diets to identify the most cost-effective interventions for meeting essential nutrient needs in populations at risk of malnutrition. These methods are based on linear programming models to identify the daily cost of a nutrient adequate (CoNA) diet, using country-specific data on availability and price matched to food composition and nutrient needs in Cost of the Diet (CotD) software (Deptford et al., 2017) updated in a new analytical platform known as ENHANCE (Koenen et al., 2022). Unlike the global data shown in Fig. 1, Fig. 2, operational analyses to target interventions may include constraints on quantities of food items per meal for different individuals, and inclusion or exclusion of specific items to align with local dietary practices (Deptford et al., 2017).

FNG analyses typically calculate and use both CoNA and CoHD, and use context-specific constraints on staple food items with a life-cycle approach to model the impact of interventions on the cost and affordability of meeting nutritional goals for each group at risk of malnutrition (Knight et al., 2024). The cost and affordability of nutrient-adequate diets is used to model impact of interventions that fill nutrient gaps, such as fortification or micronutrient supplementation, improve diversity by increasing availability and lowering cost and/or increasing income and expenditure to provide evidence on how these can increase the share of a population able to afford a nutrient-adequate diet. The cost of a nutrient-adequate diet indicator enables the assessment of the potential impact of interventions for specific groups in the lifecycle with higher nutritional needs. These estimations are especially meaningful where the gap between lowest-cost diets and food expenditure is large, and healthy and nutrient-adequate diets are out of reach for the majority of the population, with greater consequences for individuals in phases of the lifecycle with higher nutrient requirements such as during pregnancy and breastfeeding, infancy and early childhood, and adolescence. The impact of interventions to improve diversity or increase income can affect the affordability of both the nutrient-adequate and the healthy diet.

The affordability of a nutrient-adequate diet in FNG analyses is assessed subnationally, by comparing the cost of a nutrient-adequate diet with food expenditure from national household consumption and expenditure surveys. Households with food expenditure falling below the cost of the nutrient-adequate diet are deemed as unable to afford such a diet. FNG diet cost and affordability assessments are part of a broader framework and process that involves consultation with stakeholders to understand context-specific barriers to consuming diets that meet nutrient needs for individuals of different age, sex and reproductive status (Bose et al., 2019). The FNG process, which has so far been undertaken in almost 50 countries (WFP, 2023), identifies multi-sectoral opportunities and entry points that can contribute to filling nutrient intake gaps for households and individuals through the modelling of potential interventions and by convening stakeholders to review and discuss the results of the analysis. Modelling least-cost diets under different scenarios generates evidence on the potential impact of context-appropriate interventions that aim to improve access to healthy and nutrient-adequate diets, and nutrition-specific interventions such as micronutrient supplementation that help to meet the nutrient requirements of specific target groups in a population.

## 4 Use of cost and affordability indicators to inform policy and programmatic actions

Both the CoAHD suite of indicators and FNG assessments can inform policy dialogue and guide interventions. CoAHD has inspired analyses and debates about how to repurpose agricultural policy to make healthy diets more affordable (FAO, IFAD, UNICEF, WFP and WHO, 2022), focusing attention on the least expensive products in nutrient-rich food groups to show where and when improved production and distribution could improve access to healthy diets as specified in national dietary guidelines. Food system analyses have begun to model the factors influencing high cost of healthy diets (Glauber and Laborde, 2023; Laborde et al., 2021; Mekonnen et al., 2023), and FAO is using CoAHD indicators in regions to understand policy options (FAO, IFAD, PAHO, UNICEF and WFP, 2023). The cost of a healthy diet, as well as the number and percentage of people in each country unable to afford a healthy diet, are key outcome indicators in the Food Systems Dashboard that assembles indicators across the food system to illustrate possible linkages between food supply, food environments, and food security, diet, and nutrition outcomes; and which has been replicated and adopted in several countries for subnational-level information (Fanzo et al., 2020). The Food Prices for Nutrition project has undertaken technical assistance workshops in six countries (Ethiopia, Ghana, Malawi, Nigeria, Pakistan, Tanzania) to facilitate the calculation of CoAHD indicators using food price data regularly collected by the national statistical offices and other ministries, for use in policy dialogue on food security and nutrition. Similarly, the WFP team in addition to the assessment of cost and affordability of nutrient adequate diets and modeling of specific interventions to estimate their contribution to lowering cost and improving affordability of meeting nutrient needs to inform a national level dialogue on integrating nutrition across sectors in nearly 50 countries, has supported government agencies in Ethiopia, Sri Lanka and West Africa (CILSS) to use food price monitoring data to monitor cost and affordability for early warning and inform anticipatory action.

Results from the assessment of the cost and affordability of healthy diets can be used to distinguish among barriers to healthy eating. Comparing least-cost healthy diets with actual consumption can reveal where healthy diets are likely affordable but not consumed due to the many other influences on food choice, including individual-level factors such as knowledge, preferences, and time constraints, and factors in the food environment other than affordability, including availability, convenience, marketing and quality (Herforth and Ahmed, 2015). Where healthy diets and nutrient-adequate diets are unaffordable, these results can be used as a diagnostic to identify when populations face food availability constraints especially in terms of too limited diversity, too high diet costs, and/or when populations have unusually low income (Balagamwala et al., 2024). In the first two cases, policy responses might include investments to improve food production and distribution in order to lower cost and to add nutritional value (preservation, (bio)fortification), and comparison among least-cost items could help to identify which foods and food groups and value chain interventions would have the most potential to improve the affordability of healthy and nutrient-adequate diets. For affordability, diet costs could be used to assess the adequacy of livelihood interventions to improve incomes and social assistance and humanitarian food assistance transfers. Furthermore, impact on nutrition can be further increased by enhancing the nutritional value of the transfer by providing access to specific nutritious foods at lower cost to recipients of these transfers (for examples, see below). Specific country FNG assessments use both cost of nutrient-adequate and cost of healthy (i.e. diverse) diets in highlighting the issue of poor access to healthy and nutritious diets, and the impact of interventions to lower cost and to increase income. Below, we highlight results from a selection of modelled interventions from recent FNG analyses, to show how least-cost diet metrics can be used to inform and guide national and subnational responses to poor nutrition.

### 4.1 Interventions to lower the cost of nutrient-adequate diets

**Fig. 3:**
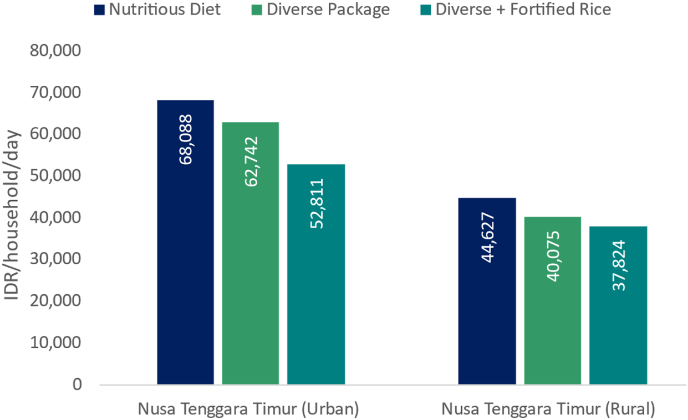
Household daily cost of the nutrient-adequate diet across SEMBAKO scenarios, Nusa Tenggara Timur, Indonesia Note: The diverse SEMBAKO package includes 10 kg of rice with the remaining value of the transfer used for eggs, tofu, chicken, banana, and cassava leaves.

Source: Figure unpublished, based on data from WFP (2021a).

Modelling is used in FNG analyses to assess the potential for interventions to improve access to nutrient-adequate diets through avenues such as lowering diet costs or increasing household food expenditure. Cost reductions for nutrient-adequate diets may be achieved through interventions that increase the availability or reduce the cost of nutritious foods, or improve the nutrient content of available foods, such as fortification. An example falling within this category is a modelled intervention aiming to make the voucher-based food assistance programme in Indonesia, SEMBAKO, more nutrition-sensitive (WFP, 2021a). FNG modelling compared various scenarios for SEMBAKO food bundles, all with equal cash value and including at least 10 kg of rice and a variable number of eggs, and varying quantities and types of additional food items purchased by recipient households. The more diverse the additional items purchased (in lieu of the value of a number of eggs), the higher the share of the nutrient-adequate diet cost the bundle represented, even though the cash value remained the same and the number of eggs decreased. The most diverse food bundle modelled included tofu, chicken, banana and cassava leaves, in addition to 10 kg rice and a number of eggs. Furthermore, the modelling results showed that substituting unfortified rice with post-harvest fortified rice in the SEMBAKO food bundles would enhance the programme's impact on nutrition and health by lowering the cost that remained for the household to be able to afford a nutrient-adequate diet further (Fig. 3) (WFP, 2021a).

**Fig. 4:**
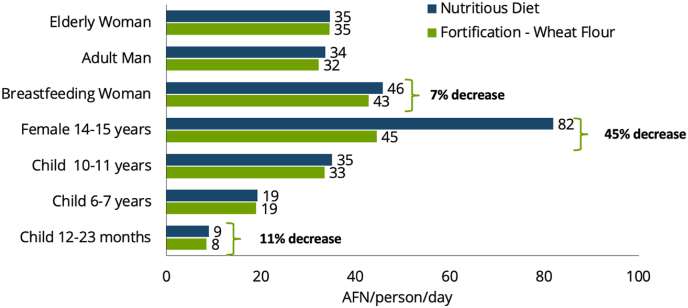
Cost of a nutrient-adequate diet by household member, urban Kabul, Afghanistan.

Source: WFP (2021b).

Similarly, modelling for Nigeria suggests that consumption of fortified rice can lower a household's cost of the nutrient-adequate diet even if the fortified rice is priced higher than unfortified rice (Federal Ministry of Finance, Budget and National Planning Nigeria and WFP, 2022). FNG analyses can also identify subpopulations for which an intervention may be particularly impactful, as illustrated by an example from Afghanistan, where the modelled impact of wheat flour fortification is greater for individuals with higher micronutrient requirements (Fig. 4) (WFP, 2021b). Interventions such as fortification reduce the cost of nutrient adequacy, captured in cost of nutrient-adequate diet assessments, but would not affect the cost of a healthy diet because the CoHD indicator is based on the cost of adequate amounts of diverse food groups. These indicators are complementary, because the cost of a nutrient-adequate diet reveals impacts of nutrient-based interventions, while CoHD highlights the need for systemic attention to investment in agricultural diversity, and both indicators reveal market connectivity and other supply side barriers to sustainable and lower-cost diverse diets.

### 4.2 Interventions to increase household income or improve safety net transfers

Nutrient-adequate diets can also be made more affordable by increasing household food expenditure through income generating activities or improved social assistance transfers. This is demonstrated by a modelling example concerning the Sustainable Food Garden or P2L (Pekarangan Pangan Lestari) programme in Indonesia (WFP, 2021a). The P2L programme aims to reduce food insecurity through the establishment of community gardens. FNG modeling simulated a 30 square meter garden with a diverse set of crops, and estimated that monetization of these crops would have the potential to offset 25%–47% of the cost of the nutrient-adequate diet.

**Fig. 5:**
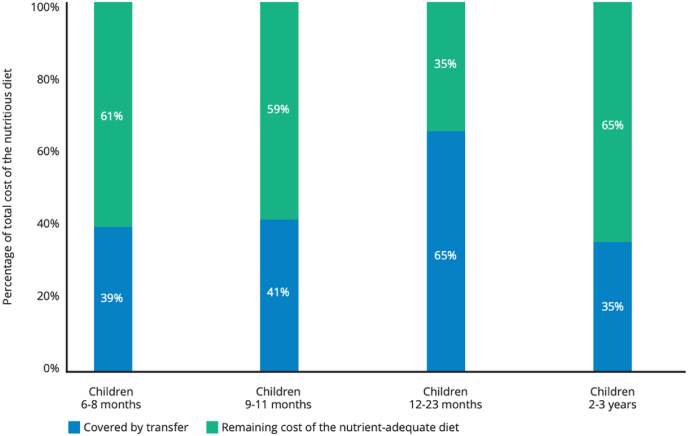
Adequacy of the UCB transfer in covering the cost of the nutrient-adequate diet for individuals of the beneficiary modelling group, average among the three modelling areas (Kisumu, Kajiado, Embu), Kenya Note: Breastfeeding is assumed for the three modelled groups with children aged between 6 and 23 months.

Source: WFP (2022).

Another example is the Universal Child Benefit (UCB) that was recently piloted in Embu, Kisumu, and Kajiado counties in Kenya, involving monthly transfers of 800 Kenyan Shillings per child under three years of age in a household (WFP, 2022). In support of the efforts by Kenya's National Social Protection Secretariat and other national and local stakeholders to advocate for the codification and national expansion of the 10.13039/100011110UCB, 10.13039/501100016038FNG modelling assessed the adequacy of the share of the 10.13039/100011110UCB transfer reserved for food to cover the cost of a nutrient-adequate diet for the beneficiary child and other household members, by applying the observed average share for food and non-food expenditure by households to the 10.13039/100011110UCB total transfer. Findings show that the transfer covers between one- and two-thirds of the child's diet cost (Fig. 5), or three to four percent of the household's diet cost.

**Fig. 6:**
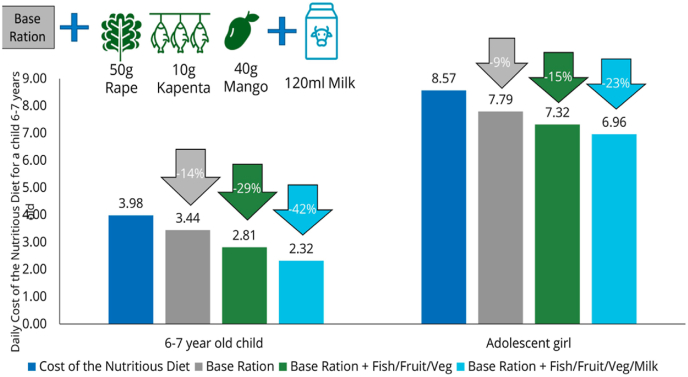
Cost of the nutrient-adequate diet for a child aged 6–7 years and an adolescent girl aged 14–15 years without and with consumption of aspirational school meals, Zambia Note: Base ration consists of 120g maize, 20g dried beans, 10g fortified oil, and iodized salt.

Source: WFP (2021c).

In Zambia, the FNG was used to investigate opportunities to improve existing school meal programmes (WFP, 2021c). Reflecting recent efforts to shift towards the use of more diverse rations, FNG modelling compared the standard base ration (i.e., 120g maize, 20g dried beans, 10g fortified vegetable oil, and iodized salt) to two alternative rations that add vegetables, fruit, and fish to the standard base ration, with and without the addition of a glass of milk. Results from this modelling suggest that diverse school meal rations containing fresh produce and animal-source products can reduce the cost of a nutrient-adequate diet of the school-going child to the household by up to 42 percent (Fig. 6), which could substantially reduce the risk of malnutrition among this age group. These results are used to inform discussions on setting nutrient targets for school meals, seeking cost-efficient ways to meet them and budget prioritization across sectors.

## 5 Conclusions

The cost and affordability of a healthy diet approach used by the FAO and World Bank and the filling the nutrient gap approach as done in the FNG analyses by the World Food Programme are complementary, showing how the identification of least-cost items and diet cost assessment helps to guide food system intervention at national and subnational levels, for the general population or for specific subpopulations. National-level estimates of the cost and affordability of a healthy diet support global efforts to track progress across countries towards Sustainable Development Goals (SDG), particularly SDG 2 (*End hunger, achieve food security and improved nutrition, and promote sustainable agriculture*), while the 10.13039/501100016038FNG approach provides a country level, sub-national update and identifies context- and population-appropriate interventions to improve access to healthy and nutrient-adequate diets.

New software tools allow users to compute the cost of healthy and nutrient-adequate diets in Excel or Stata (Food Prices for Nutrition, 2023b) or in platforms relying on algorithms programmed in Delphi or dot net (Deptford et al., 2017) or Python (Koenen et al., 2022), using their own food price data and dietary guidelines. For example, the Ethiopian Public Health Institute (EPHI) has pioneered national monitoring of the cost and affordability of a healthy diet since July 2023 (Alemayehu et al., 2023) using the Ethiopian food-based dietary guidelines (Federal Government of Ethiopia, Ministry of Health, Ethiopian Public Health Institute, 2022) and CPI price data from the Ethiopian Statistical Service. The EPHI has also used these price data to monitor the cost and affordability of nutrient-adequate diets calculated using the CotD software (Ethiopian Public Health Institute and WFP, 2022, 2021).

Regular publication of healthy diet costs as part of a country's official national statistics was first done by Nigeria, using item availability and price collected for their consumer price index (National Bureau of Statistics, 2024). Enhanced availability of high-quality food price data, matched to food composition data and dietary requirements, would improve the generation of least-cost benchmark diets in terms of their frequency, precision, recency, and geographic specificity. These least-cost healthy and nutrient-adequate diets can be used in turn to identify effective nutrition-sensitive policies across sectors, assess and improve food security, and guide food system transformation.

## Funding

William A. Masters, Anna Herforth and Yan Bai report financial support provided by 10.13039/100000865Bill & Melinda Gates Foundation and the Foreign Commonwealth & Development Office, through INV-016158. Jessica Wallingford reports financial support from a Friedman Nutrition and Citizenship Fellowship from 10.13039/100008090Tufts University, and a Social Sciences and Humanities Research Council Doctoral Fellowship from the 10.13039/501100000023Government of Canada.

## CRediT authorship contribution statement

**Jessica K. Wallingford:** Visualization, Writing – original draft, Writing – review & editing. **Saskia de Pee:** Conceptualization, Writing – review & editing. **Anna W. Herforth:** Conceptualization, Writing – review & editing. **Sabrina Kuri:** Conceptualization, Writing – review & editing. **Yan Bai:** Writing – review & editing. **William A. Masters:** Conceptualization, Writing – review & editing.

## Declaration of competing interest

The authors declare the following financial interests/personal relationships which may be considered as potential competing interests: Jessica Wallingford reports financial support was provided by a Friedman Nutrition and Citizenship Fellowship from Tufts University and by a Social Sciences and Humanities Research Council Doctoral Fellowship from the Government of Canada. William A. Masters reports financial support was provided by 10.13039/100000865Bill & Melinda Gates Foundation and the Foreign Commonwealth & Development Office, through INV-016158. Anna Herforth reports financial support was provided by 10.13039/100000865Bill & Melinda Gates Foundation and the Foreign Commonwealth & Development Office, through INV-016158. Yan Bai reports financial support was provided by 10.13039/100000865Bill & Melinda Gates Foundation and the Foreign Commonwealth & Development Office, through INV-016158. If there are other authors, they declare that they have no known competing financial interests or personal relationships that could have appeared to influence the work reported in this paper.

## Data Availability

Data are publicly available from the Cost and Affordability of a Healthy Diet database in FAOSTAT and the World Bank’s Food Prices for Nutrition Database, version 2.0.
